# Insights on the Influence of Sugar Taxes on Obesity Prevention Efforts

**DOI:** 10.1007/s13668-019-00282-4

**Published:** 2019-06-08

**Authors:** Melissa A. Fernandez, Kim D. Raine

**Affiliations:** School of Public Health, University of Alberta, 4-077 Edmonton Clinic Health Academy, 11405 – 87 Ave., Edmonton, AB T6G 1C9 Canada

**Keywords:** Obesity, Policy, Sugar-sweetened beverages, Tax, Public health

## Abstract

**Purpose of Review:**

This review will present the latest evidence on the impacts of sugar taxes on obesity with a focus on sugar-sweetened beverages (SSB).

**Recent Findings:**

Evidence of direct impacts of SSB taxation policies on obesity prevalence continues to be limited. Natural experiments involving SSB taxation policies implemented in Mexico and Berkley, CA, indicate that this type of intervention alters beverage consumption patterns. Naturalistic evidence in combination with modeling studies suggests that SSB taxation is a viable anti-obesity policy. However, researchers and public health practitioners need to be vigilant of industry tactics to curtail SSB lowering efforts.

**Summary:**

To maximize the impacts of SSB taxation, it should be combined with interventions that increase access to non-sweetened beverages, educate consumers about alternative healthy beverages, and explore taxation of other non-nutritive foods and beverages. Furthermore, both intended and unintended consequences of interventions should be closely monitored.

## Introduction

As of 2015, an estimated 107.7 million children and 603.7 million adults around the world had obesity [1]. The prevalence of obesity among adults is increasing worldwide [2], which is a driver for other diet-related non-communicable diseases such as cardiovascular disease and diabetes. Equally concerning, severe obesity, defined as BMI ≥ 35, or BMI > 120% of the 95th percentile for age and sex among children is on the rise [3]. There have been concomitant increases in obesity prevalence and the consumption of ultra-processed foods including sugary foods and drinks [4–8]. Ultra-processed food and drink consumption, accounting for the majority of sugar intake in the diet, is associated with poor diet quality and excess energy intake [9–11]. Poor diet is a leading risk factor for non-communicable diseases [12]. There is substantial evidence indicating that reducing consumption of highly processed products will reduce the risk of developing diet-related diseases [13]. To diminish the health and economic burden of diet-related diseases, there is a clear need to address the prevalence of obesity and improve dietary patterns among the public. The use of economic tools to address food affordability and purchase incentives is one of the policy interventions recommended to promote healthy diets and reduce obesity that was outlined in the World Cancer Research Fund International’s NOURISHING framework [14]. Taxing sugar-sweetened beverages (SSBs) is one such economic tool.

## Rationale for Sugar Taxes

Strong support for fiscal policies is based on the rationale that modifying the price of a product can alter its consumption [15]. Additionally, increasing the price of SSBs could reduce the price gap with healthier beverages such as milk, encouraging individuals to select healthy alternatives more often [16]. The simple price manipulation of foods can alter consumption patterns in such a manner that could ultimately reduce the development of diet-related diseases [17]. The 2016 World Health Organization’s Report of the Commission on Ending Childhood Obesity listed a package of six comprehensive recommendations to address childhood obesity. The first recommendation was to: “implement comprehensive programs that promote the intake of healthy foods and reduce the intake of unhealthy foods and sugar-sweetened beverages by children and adolescents”, which includes a tax on SSBs [18]. The choice to target SSB taxes as health policy is based on (1) their strong link to obesity, (2) they are inherently non-nutritive, and (3) they have high price elasticity.

There is mounting evidence of a causal link between sugar added to beverages and increased risk for non-communicable diseases [19]. Sugar-sweetened beverage (SSB) consumption remains very high across the globe and they are the largest source of added sugar in the American diet [20, 21]. A single serving of 330 mL or 500 mL can provide between 72% and 104%, respectively, of the total maximum daily calories from sugar (10% of daily kcal or ~ 50 g) recommended by the WHO [22]. SSBs contain excessive amounts of energy, in the form of simple sugar, that do not provide any health benefits. Unlike eating foods, there are no compensatory mechanisms after drinking beverages to mitigate the excess energy intake from SSBs, potentially leading to excess weight gain [23–25]. Finally, the high price elasticity of SSBs means that the reduction in SSB consumption would mirror the increase in its price, making SSBs an ideal product for taxation [26, 27, 28••]. For example, a 10% increase in price is expected to reduce SSB consumption by approximately 10% ranging from a 7% reduction in SSBs for infrequent SSB consumers to as high as a 17% reduction among the highest consumers [28••].

## Prevalence of SSB Taxes

**Fig. 1:**
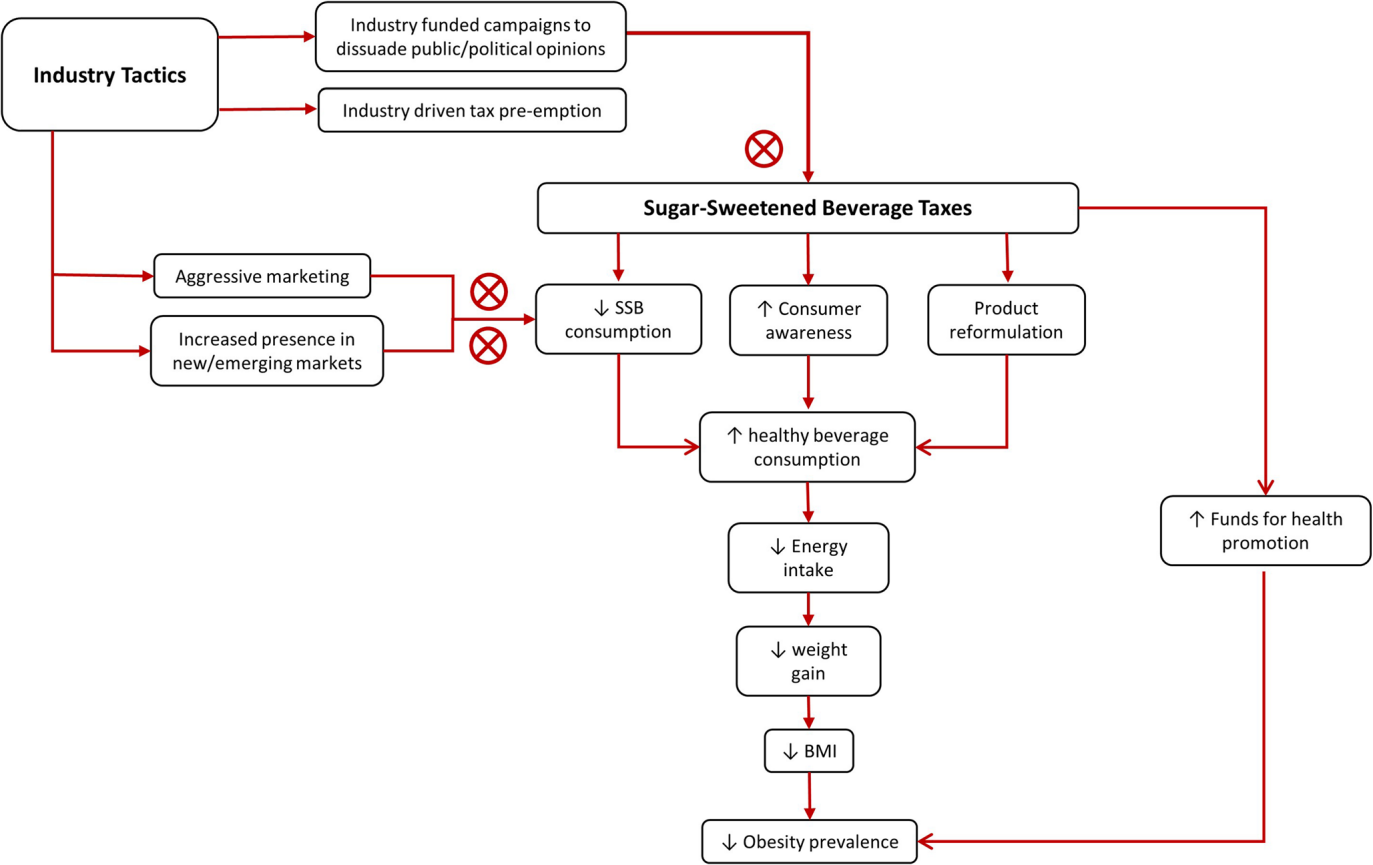
Direct and indirect influences of sugar-sweetened beverage (SSB) taxes on reducing obesity prevalence

Sugar taxes were documented as early as the 1920s and 1930s in countries like Norway and Denmark, implemented strictly as a fiscal measure to generate revenue [29]. There has been a proliferation of SSB taxes with implementation in over 40 new settings around the world in less than 10 years. In 2016, Le Bodo et al. inventoried 22 separate cases of SSB taxes dating from 2002 that represented country-, state-, and city-level fiscal policies [30]. Overall, these more recently implemented SSB taxes have been as much a fiscal policy to generate general government revenue as a health policy to reduce consumption of SSB. Funds raised through taxation are also increasingly dedicated to intensify health promotion efforts [30]. Therefore, the complex policy changes needed to implement an SSB tax should be considered a collaborative cross-ministerial effort [31]. As a health policy to address non-communicable diseases (NCDs) by reducing sugar consumption, SSB taxes are largely supported by nutrition experts and international health organizations [13, 18, 20, 32]. Not only are taxes expected to decrease SSB consumption, leading to a decrease in obesity and NCDs, but they are also expected to drive the food industry towards product reformulation by decreasing sugar content, increase public awareness about high sugar consumption, and generate revenue that can be reinvested in health and social programs (Fig. 1) [22].

## Evidence Supporting Sugar Taxes

While sugar taxes are not new, monitoring their impacts on diet and health is relatively recent. With little available data, it is still early to judge their influence on distal population-level health outcomes such as NCD. Nevertheless, the decreased sale of SSB in locations where taxes have now existed for several years [33••, 34, 35••] infers potential for long-term positive health impacts. Furthermore, long-term benefits on diet-related disease prevalence can be potentiated by investing SSB tax revenue into health promotion programs [36•]. While there is still little evidence of an impact of sugar taxes specifically on obesity, there is growing evidence from naturalistic studies in regions where taxes have been implemented that monitor both purchasing habits and health outcomes to support modeling studies, building the case for the continued proliferation of SSB tax implementation [37•].

### Modeling and Observational Studies

In 2013, a systematic review identified nine articles between published between 2000 and 2013 that examined the effects of fiscal policies (taxes and price increases) on body mass index (BMI) and weight status [27]. The review identified six modeling studies on BMI, overweight or obesity, all from the USA, with a wide range of methodologies examining various populations (children, women, men, and adults) in different settings [38–44]. The mix of study designs and populations made it difficult to draw firm conclusions about the effects of taxes or price changes on adiposity, but positive conclusions from numerous studies led review authors to conclude that SSB taxation could reduce obesity [27].

A retrospective cohort study of 6537 men and 5324 women who participated the Monitoring the Future Surveys (1992–2003) determined that a $1 increase in price of a 2-L bottle of SSB was associated with a reduction in the probability of obesity of 28.1% and 10.8% in women and men, respectively [41]. Among children and adolescents, using NHANES data from 1999 to 2006, a 1% increase in SSB prices was estimated to result in a minute reduction in obesity prevalence by 0.009% [39]. Other modeling studies have found similar conclusions with slight decreases in BMI associated with a 1% increase in SSB prices [38, 43, 44]. American food consumption data from NHANES 2003–2006 were used to model a tax-induced 20% increase in price and calculated a decrease in calorie intake from SSBs by 54.6 kcal/day with a concurrent increase in calories from juices by 12.5 kcal/day. These changes in beverage intake represented a net reduction of 39.5 kcal/day, equivalent to a reduction in weight of 1.9 kg per year [42]. Using data from a single year of the Nielsen Homescan panel, a 20% and 40% tax on SSB was expected to reduce weight by 0.32 kg and 0.59 kg per year, respectively, while generating considerable revenue [40]. A Canadian simulation modeling study predicted that over 25 years, a 20% tax on sugary drinks would prevent over 700,000 cases of overweight and obesity, and over 200,000 cases of type 2 diabetes, saving $11.5 billion (CAD) in direct health care costs and generating $1.7 billion [45]. However, it is impossible to predict how the industry will respond to SSB taxation (e.g., intensify marketing, increase sales in non-taxed markets, or reformulate products) or how consumers will modify their beverage consumption (e.g., substitute taxed SSBs with cheaper sweetened alternatives or select healthier untaxed alternatives).

## Natural Experiments

Though SSB taxation has been documented for over a decade, to date, the impacts of a tax on SSBs have only been evaluated in a handful of settings. Within the last 5 years, the implementation of SSB taxation in Mexico, the city of Berkeley, California, the states of Maine and Ohio in the USA, and in Barbados has laid the groundwork for evaluations studies. Natural experiments are possible in settings where pre-tax sales and consumption data are available and post-tax data are closely monitored for comparison.

### Mexico

In response to high obesity and diabetes rates, in January 2014, Mexico implemented a specific excise tax (1 peso/L) on non-alcoholic beverages with added sugars, which represent an approximate 11% increase in the price of carbonated sweetened beverages. Within the first year of the tax, there was a marked monthly purchase reduction in taxed SSBs, reaching 12% by December 2014 and averaging a reduction of 6% over 2014. The decrease was the highest in groups with low-socioeconomic status reaching 17% in December. There was an increase in purchases of untaxed beverages of 4% over the same time period, mainly driven by bottled water [46]. Sales data showed similar trends: a 7.3% decrease in per capita SSB sales and a 5.2% increase in plain water sales [47]. Over a 2-year span, following the implementation of the tax, purchasing of taxed beverages decreased by 9.7% and the group with the lowest socioeconomic status still had the highest decrease in purchasing taxed beverages. A sustained decrease in SSB purchasing coincided with SSB tax implementation [33••]. Similarly, the tax on energy-dense non-essential packaged foods implemented alongside the SSB tax in Mexico resulted in a 5.1% decrease in taxed foods purchased. The decrease was the highest among the group with the lowest socioeconomic status (10.2%), lower in the medium socioeconomic group and there was no change in the high socioeconomic group [48]. Concomitant interventions to tax SSBs and snack foods will likely have a synergistic impact on improving dietary patterns, potentiating long-term effects on health outcomes.

### Maine, Ohio, and Berkeley, California

The state of Maine implemented a sales tax of 5.5% on soft drinks in 1991, which was reduced to 5% in 2001 and applied to snack foods and carbonated water. Ohio implemented a sales tax of 5% exclusively to soft drinks in 2003. In both states, the sales taxes did not alter consumption or sales patterns, although they did generate substantial general revenue [49]. It is possible that a tax of 5% is not substantial enough to deter purchasing or consumption, particularly when the tax is not widely known and revenue is not reinvested into health programming. The city of Berkeley in California implemented an excise tax of $0.01 USD per fluid ounce on SSBs in March 2015, becoming the first US jurisdiction to implement an excise tax (i.e., applied at the manufacturer or merchant level by product weight or volume). Pre- and post-sales consumption data in low-income neighborhoods was compared to neighboring cities of Oakland and San Francisco, California. Data were collected via a beverage frequency questionnaire 8 months prior to voting for the tax and 4 months after implementation. There was a substantial decrease (21%) in SSB consumption in Berkley in contrast to a moderate increase (4%) in Oakland and San Francisco. Additionally, water consumption increased by 63% in Berkeley and by 19% in neighboring cities [50]. When taxes are implemented in a single city, there is potential for individuals to travel outside the taxed area to make their usual purchases. However, it is also plausible that the intervention in Berkeley created awareness in neighboring cities, thereby indirectly influencing consumption behaviors in other jurisdictions. A year after implementing the excise tax in Berkeley, the impacts on beverage prices, sales, store revenue/consumer spending, and usual beverage intake were examined. Sales of SSBs declined by 9.6% in Berkeley, whereas they increased by 6.9% in non-Berkeley stores. Additionally, non-taxed beverage sales increased by 3.5% in Berkeley and 0.5% in non-Berkeley stores, driven mainly by water. Transactions were on average $0.18 USD less after the tax. There were no significant reductions in SSB intake or per capita SSB caloric intake; however, baseline levels of SSB intake were already much lower than the national average (45 kcal/day compared to 131 kcal/day). These results indicate that SSB taxes can be effective in influencing healthier beverage purchases and do not impose undue financial hardship on consumers [34].

### Barbados

In September 2015, Barbados implemented a 10% excise tax on SSB. Evaluation of price increases found a divergence in the prices of taxed and untaxed beverages. There was an average price increase of 5.9% in taxed beverages with a slight dip of < 1% in untaxed beverages. This divergence may be a response from industry to drop prices of untaxed beverages [51]. An interrupted times series study design was used to assess changes in beverage sales from January 2013 (prior to SSB tax) to October 2016 (1-year post-tax implementation). On average, weekly sales of SSBs decreased by 4.3% while non-SSBs increased by 5.2%. However, there was evidence of an increase in sales of cheaper SSBs, indicating that individuals may be substituting high-cost taxed SSBs for lower-cost taxed SSBs. The extent of downshifting from brands that are more expensive to less expensive brands in response to higher prices needs further exploration [35••].

Overall, results from natural experiments generally support predicted reductions in SSB purchases that are presumed to relate to decreased consumption demonstrated in modeling studies and are extremely promising. However, an extended evaluation period will be needed to adequately monitor and evaluate the long-term impacts of SSB tax implementation on obesity. Furthermore, impacts on nutrition indicators (diet quality and dietary patterns) should be examined in conjunction with adiposity measures.

## Challenges

Despite the growing popularity of fiscal measures to improve diet and health outcomes, food taxes have garnered mixed public opinion and prompted a backlash from the food industry. There have been cases of policies being repealed, slowed down, or even blocked before implementation. Public support necessary to influence decision-makers’ adoption of fiscal policy may not always be favorable. Concerns include the following: objections to the government interfering with the market, the argument that taxes are an overly simple solution to a complex problem so will not solve obesity, and objections to use of tobacco taxes as exemplars in that unlike tobacco, we need food to live [52]. However, counter-arguments include that taxes are not meant to be a solution in isolation, but part of a larger portfolio of policy measures (restrictions on advertising of unhealthy products, healthy food policies in public spaces), as was the case in tobacco control. Tax regressivity, a greater financial burden for lower income groups, is a major preoccupation of the public [17], particularly when other interventions are not implemented (e.g., healthy food subsidies) to mitigate perceived unintended effects. Despite this concern, food taxation schemes appear to confer the greatest benefits among lower socioeconomic groups [33••, 34, 48, 51].

Public opinion surveys reveal that restrictive fiscal policies such as taxation are less popular than non-restrictive policies such as providing nutrition information of front of the package. For example, in Australia, 89.7% of the public was in favor of mandatory front-of-pack nutrition labels compared to only 41.9% in favor of a tax on SSB with the strongest opposition coming from the most disadvantaged groups [53]. In Canada, surveys of policy influencers revealed 57% support for taxes on foods and beverages, while support for public nutrition education received almost universal support (99%) [54]. In two other Australian studies, support for taxation increased when intentions to dedicate revenue to health initiatives were made clear [55, 56]. Dedicating at least 50% of revenue generated to programs that support health has been recommended [16]. While the World Health Organization has recommended that Member States consider taxing not just SSB, but all energy-dense foods and beverages, there may be numerous logistic and legal barriers with taxing foods from heterogeneous categories of foods that contain some nutritive value. For example, Denmark repealed a tax on saturated fat a year after it was implemented and canceled plans to implement a tax on foods with added sugar, in part because of lack of public support [57].

Another major challenge to implementing new sugar taxes are industry tactics that are used to dissuade the public and pressure governments into rescinding plans for taxation (Fig. 1) [58]. Food industry lobbyists are prominent at the state level in the USA and seem intent on pre-empting new local taxes from being implemented across the country [59]. Additionally, the effects of taxes may be neutralized if they use tactics such as aggressive marketing, increasing sales in untaxed regions, and negative public education campaigns that cast doubt about the intentions of a sugar tax [58]. Health agencies and public advocacy groups need to be prepared and anticipate how to react when the industry aggressively opposes actions towards passing SSB taxes [58].

It is clear that a well-designed fiscal policy to tax sugary foods and beverages can have significant impacts on price and therefore purchasing and consumption behaviors [17, 28••]. However, the impacts of sugar taxes on improving dietary intakes and more distal health outcomes such as obesity are not yet clear. Only a few settings with implemented taxes have conducted evaluations. The lack of monitoring may indicate a larger problem related to capacity and readiness to monitor changes in the food environment or suggest that the taxes were economically motivated rather than driven by health needs. In the coming years, strong evidence will be needed to justify the maintenance of current taxes as well as expand taxes to other food and beverage categories. Monitoring the impacts of current fiscal interventions is therefore crucial. Settings considering implementing new taxes should plan for the inclusion of robust impact evaluations by monitoring pre- and post-tax sales, consumption data, diet quality, adiposity, and NCD rates over several years [32].

## Conclusion

No single intervention in isolation is likely to have significant impacts on population-level health outcomes such as reducing the prevalence of obesity and associated NCD. Combined and persistent efforts that act synergistically to influence short-term purchasing patterns, dietary practices, and longer-term health outcomes will be necessary. The full potential of SSB taxes to improve health outcomes will likely only be demonstrated in settings that implement fiscal policies alongside non-tariff interventions such as behavior change campaigns and increasing the availability and/or affordability of healthy beverage alternatives. Like all policy interventions, when they are combined with multisector and multilevel supports, better outcomes can be expected.

As more countries consider adopting SSB taxes, impact evaluations should be integrated into policy planning to ensure timely collection and capacity for close monitoring of effects on diet and health. These data are needed to ensure continued justification of taxation in addition to building a case for expanding fiscal measures to other non-nutritive food and beverage categories. There are evident research gaps that can be addressed in terms of how sugar taxes can work in concert with other interventions and monitoring SSB intakes in different population subgroups. Furthermore, understanding how to adapt the intervention to local contexts as well as to foresee potential challenges from industry will facilitate implementation and impact.
